# Generation and characterization of a human single-chain fragment variable (scFv) antibody against cytosine deaminase from Yeast

**DOI:** 10.1186/1472-6750-8-68

**Published:** 2008-09-10

**Authors:** Alessandra Mallano, Silvia Zamboni, Giulia Carpinelli, Filippo Santoro, Michela Flego, Alessandro Ascione, Mara Gellini, Marina Tombesi, Franca Podo, Maurizio Cianfriglia

**Affiliations:** 1 Department of Therapeutic Research and Medicines Evaluation, Istituto Superiore di Sanità, Viale Regina Elena 299, Rome, Italy; 2 Department of Cell Biology and Neurosciences, Istituto Superiore di Sanità, Viale Regina Elena 299, 00161 Rome, Italy

## Abstract

**Background:**

The ability of cytosine deaminase (CD) to convert the antifungal agent 5-fluorocytosine (5-FC) into one of the most potent and largely used anticancer compound such as 5-fluorouracil (5-FU) raised considerable interest in this enzyme to model gene or antibody – directed enzyme-prodrug therapy (GDEPT/ADEPT) aiming to improve the therapeutic ratio (benefit versus toxic side-effects) of cancer chemotherapy. The selection and characterization of a human monoclonal antibody in single chain fragment (scFv) format represents a powerful reagent to allow in *in vitro *and *in vivo *detection of CD expression in GDEPT/ADEPT studies.

**Results:**

An enzymatic active recombinant CD from yeast (yCD) was expressed in E. coli system and used as antigen for biopanning approach of the large semi-synthetic ETH-2 antibody phage library. Several scFvs were isolated and specificity towards yCD was confirmed by Western blot and ELISA. Further, biochemical and functional investigations demonstrated that the binding of specific scFv with yCD did not interfere with the activity of the enzyme in converting 5-FC into 5-FU.

**Conclusion:**

The construction of libraries of recombinant antibody fragments that are displayed on the surface of filamentous phage, and the selection of phage antibodies against target antigens, have become an important biotechnological tool in generating new monoclonal antibodies for research and clinical applications. The scFvH5 generated by this method is the first human antibody which is able to detect yCD in routinary laboratory techniques without interfering with its enzymatic function.

## Background

The ability of cytosine deaminase (CD) to convert the clinically used antifungal agent 5-fluorocytosine (5-FC) into one of the most potent and largely used anticancer agent such as 5-fluorouracil (5-FU) raised considerable interest in this enzyme to design innovative anticancer therapies [1,2]. Therefore, CD-based enzyme/prodrug strategies are under investigation to model gene or antibody directed enzyme-prodrug therapy (GDEPT/ADEPT) for achieving high local concentration of 5-FU without significant systemic toxicity [3,4]. In in vivo animal model, the CD gene/enzyme which is not naturally expressed in mammals are first introduced into the cells of a tumour by specific antibodies [5-7], modified microorganisms such as bacteria and viruses or synthetic vectors (reviewed by Springer et al., 2007)[4]. When the discrimination between tumor and normal tissue enzyme levels is sufficient, 5-FC is given i.v., which is converted into 5-FU by CD within the tumor [8]. A convincing demonstration that such a complex system can be developed for clinical use requires evidence that each of the components of the gene/antibody complex functions by the mechanisms proposed [9]. This can be provided by well defined measurements including the concentration levels of the antibody-enzyme conjugate or *de novo *expressed enzyme, in plasma, tumor and normal tissues [10-12]. To allow the detection of CD expression at the protein level, we raised a human monoclonal antibody in single chain fragment (scFv) format against a recombinant CD from yeast (yCD) proved to be functionally active in NMR and in *in vitro *studies to convert the antifungal drug 5-FC into the anticancer compound 5-FU. The specificity of the human scFv was confirmed by Western blot and ELISA analyses. With this antibody, yCD expression can now be monitored without interfering with its enzymatic function in GDEPT, ADEPT and other studies leading to the effect of the so called tumour amplified protein expression and targeting (TAPET) to localize in vitro and in vivo generation of the anticancer agent 5-FU [4].

## Results and discussion

The CD/5-FC-based GDEPT or ADEPT are among the most studied strategies aiming to improve the therapeutic ratio (benefit versus toxic side-effects) of cancer chemotherapy. CD has the ability to deaminate the non toxic prodrug 5-FC into the highly toxic compound 5-FU. By inhibiting DNA synthesis this drug preferentially kills tumour cells. However, 5-FU has high gastrointestinal and hematological toxicities [2]. In contrast, the prodrug 5-FC is fairly nontoxic [13]. and CD is not naturally expressed in mammalian cells. Thus, the selectively guided CD/5-FC complex should minimize the toxic effects of 5-FU because the conversion of 5-FC to 5-FU should only occur within the tumor.

A convincing demonstration that this strategy can be developed for clinical use requires knowledge of specific parameters which may include the in in vivo monitoring of the CD complex. For this reason we have firstly constructed a novel expression system for the production of a functionally active yCD. Subsequently a fully human antibody in scFv format not interfering with yCD activity was developed and analyzed.

### Expression and purification of yCD protein

**Figure 1:**
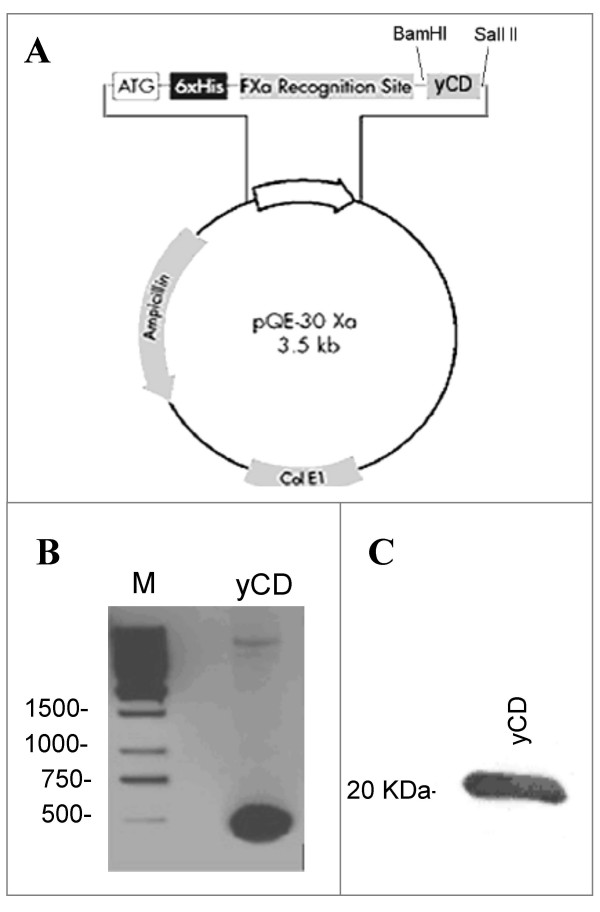
**Expression of recombinant yCD**. In (A), is depicted a schematic representation of yCD expression vector, constructed by inserting the coding sequence for yCD into pQE30Xa plasmid, and expressed in TG1 strain of *E. coli*. In (B) and (C) are shown respectively, the PCR-DNA fragment corresponding to the expected 500 bases pair encoding for yCD and the immuno-blot of the purified yCD protein.

A functionally active yCD was generated by recombinant DNA technology. The gene encoding for yCD was amplified and inserted into the pQE30Xa expression vector which contained the *lac *promoter for protein induction and 6 × His TAG sequence for purification (Fig. 1A). 500 base pairs band shown in Figure 1B corresponded to DNA fragment encoding for yCD obtained by PCR using specific primers. After TG1 E. *coli *bacterial strain transformation, several clones were isolated and proved suitable for yCD production. The clone exhibiting the best protein induction was further characterized. The yield of purified protein was about 10 mg l^-1^, using metal chelate affinity chromatography. The reliability of this novel expression system used for protein isolation and purification was confirmed by biochemical investigation showing that yCD migrated at the expected molecular weight of about 20 kDa (Figure 1C).

### Selection and characterization of scFvH5 antibody specific for yCD

**Figure 2:**
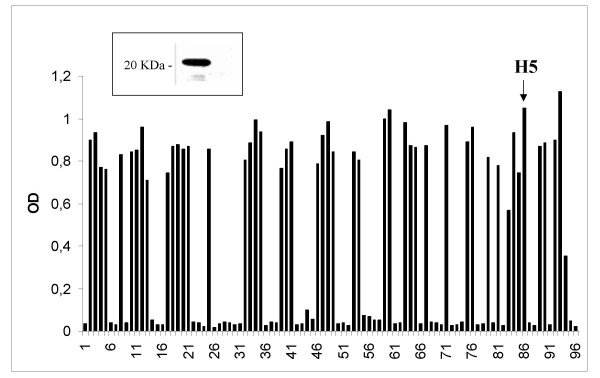
**Selection of yCD-specific scFvs**. IPTG inducted bacterial supernatants of individual colonies from the third round of the ETH-2 selection on yCD protein, were tested by ELISA in 96-well microtiter plates coated with the antigen. OD values higher than three fold the value of negative control are scored as positive. Negative and positive controls positioned in wells 1–4 reacted as expected. In the inserted box, the Western blot of yCD protein detected by scFvH5 (one of the most reactive clones) is shown.

To isolate phage-displayed specific antibodies, an aliquot of the human synthetic ETH-2 library containing approximately 1 × 10^12 ^cfu phages was panned into Nunc-immunotubes coated with 10 μg ml^-1^of purified yCD. Non-specifically absorbed phages were removed by intensive washing. Specific bound phages were eluted, amplified and used for next panning as previously described [14]. By using this protocol, we were able to isolate a phage-antibody population specifically recognizing yCD protein after only three rounds of selection. Plating on agar of TG1 cells infected with a pool of phage antibodies from third selection allowed individual clones harboring phagemid to grow. Soluble scFvs derived from IPTG inducted colonies, were screened by ELISA and several of them proved to be specific for yCD protein (Figure 2). One of the most reactive scFv antibody clone, named H5, was isolated and further characterized under biochemical and genetic aspects.

**Figure 3:**
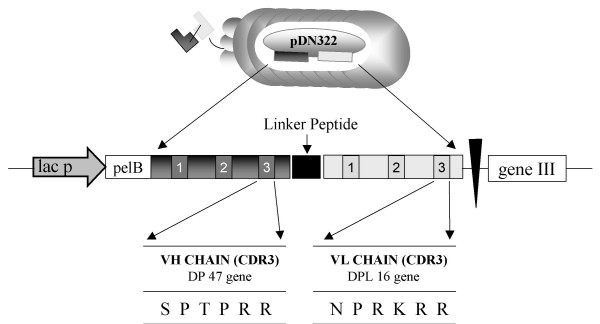
**Sequence analysis of scFv H5 and genetic structure of phage antibody from ETH-2 library**. The amino acid sequence of the CDR3 regions of the selected scFv H5 antibody are reported. A schematic representation of the scFv antibodies dislpayed on M13 phage as pIII fusion proteins is depicted.

Western blot studies showed that scFvH5 recognizes a protein band of about 20 KDa corresponding to the expected molecular weight of the purified yCD protein (see Fig. 2, inserted box). The genes encoding for variable regions of heavy (VH) and light (VL) chains of the scFvH5 were sequenced, and their corresponding amino acid aligned (Fig. 3) according to Pini et al., [15].

### Determination of yCD activity

**Figure 4:**
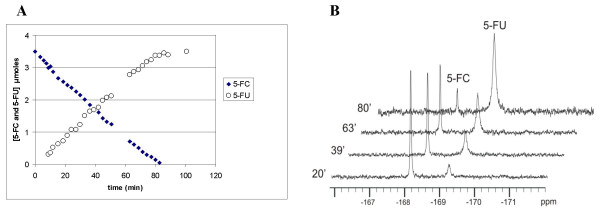
**Functional analysis of yCD by ^19^F NMR study**. In (A) and (B) are shown respectively, the 5-FU formation (μlmoles) due to the conversion of 5-FC by yCD and representative spectra during the reaction at 20, 39, 63 and 80 min.

In order to determine the functional activity of the recombinant yCD, the ability of the enzyme to deaminate 5-FC was assessed by fluorine NMR. This approach allowed simultaneous detection of the substrate and the product without interference by other compounds. Figure 4 shows that after 90 min 5-FC was completely converted into 5-FU in the presence of the yCD. Absolute quantification of the product was obtained by adding a known amount of 5-FU to the reaction mixture at the end of the experiment.

**Figure 5:**
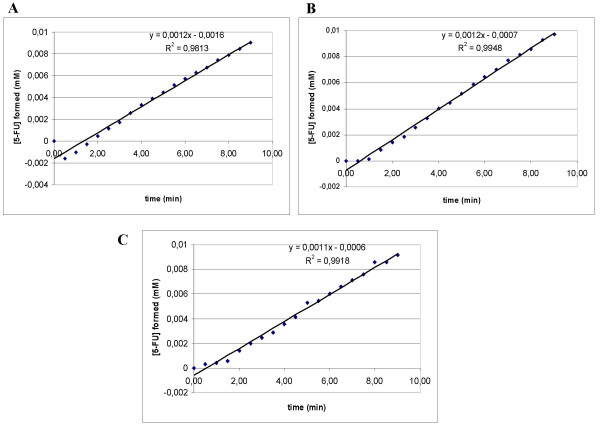
**Spectrophotometry of yCD activity**. In (A), are reported the values of de novo formed 5-FU (mM) obtained in presence of yCD (0.5 μg ml^-1^) and 5-FC (0.18 mM) during the first 9 min of the reaction. In (B) and (C) are reported the 5-FU values obtained with identical reagents but in presence of 2 μg ml^-1 ^of the specific (scFvH5) or irrelevant (scFvGO) antibodies. Slope of lines represents starting speed of the reaction. Correlation coefficient (R) indicates the strength and direction of the linear relationship between time and formed 5-FU.

The specific yCD enzymatic activity was also assessed by spectrophotometric analysis in order to determine nanomolar concentrations of the reaction product. Figure 5A shows the initial velocity of the reaction which is represented by direction coefficient of the line plotted placing concentration of formed 5-FU versus reaction time.

In order to assess if the enzymatic activity of yCD was affected by the presence of the scFvH5 an identical experiment was performed in presence of the antibody. Figure 5B shows that the rate of product formation was similar to that with free yCD, suggesting that there was no apparent loss in enzyme activity as a result of binding with scFvH5. Identical results were obtained using the irrelevant scFvGO antibody (see Figure 5C).

### Cytotoxic assay

**Figure 6:**
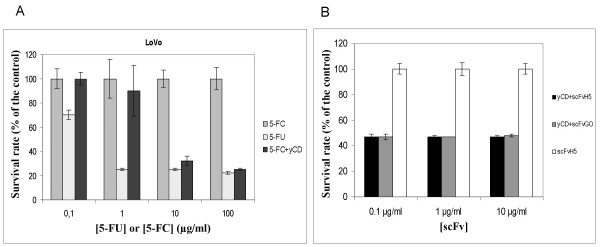
**In *in vivo *assay of yCD protein**. In (A), LoVo cells were seeded in 96-well plate (2500 cells/well) and cultured in BM for 4 days containing 2.5 μg ml^-1 ^of yCD in presence of the indicated concentrations of 5-FC. In (B), the cells were culture at same conditions but in BM containing 2.5 μg ml^-1 ^of yCD and 10 μg ml^-1 ^of 5-FC in presence of different concentrations of scFvH5 or the irrelevant scFvGO antibodies. Cell cytotoxicity (due to *de novo *formed 5-FU) was evaluated by WST-1 assay and calculated as a percentage of survived cells. Values are reported as the mean of triplicate samples. The bars indicate SD.

Using an *in vitro *model constituted by human LoVo cells, we measured the enzymatic activity of the recombinant yCD protein in converting the antifungal agent 5-FC into the highly toxic anticancer compound 5-FU. In parallel we evaluated if co-incubation of the same reagents with scFvH5 affected yCD function. Figure 6A shows that 2.5 μg ml^-1 ^of yCD exerted a significative cell growth inhibition of the human carcinoma LoVo cells in the presence of 5-FC concentration ranging from 1 mg ml^-1 ^and 10 μg ml^-1^. In contrast, the co-incubation of yCD and 5-FC with various concentration of scFvH5 did not interfere with the cytotoxic activity of *de novo *generated 5-FU (Figure 6B).

The results above reported demonstrated that, yCD produced by the novel expression system here described acts as an active enzyme in converting 5-FC into the anticancer compound 5-FU. Moreover, the binding of the human scFvH5 with yCD did not affect the enzyme function. In particular, our studies demonstrated that the presence of scFvH5 did not interfere with yCD in converting 5-FC or with the cytotoxic activity of *de novo *formed 5-FU.

## Conclusion

The monoclonal antibody scFvH5 may be a very useful reagent for detection of CD expression in GDEPT/ADEPT studies. In fact, this mAb detects functional yCD either in ELISA or in Western blot studies (Figure 1 and 2) thus providing evidence that similar techniques may be extended to measure yCD levels in plasma, tumor and normal tissue samples. Since its particular genetic origin, the scFvH5 can be easily genetically engineered to construct a whole human antibody with a predefined IgG subclass, for selective removal of mAb-yCD conjugate from the circulation, without interfering with the enzyme function.

Differently with other mAbs to CD generated by hybridoma [5] or recombinant DNA technologies [16], the scFvH5 is the first fully human monoclonal antibody in scFv format so far described which is able to detect yCD protein in different routinary laboratory techniques. Hence, this antibody may represents an excellent candidate for in vivo detection and measurement of the CD complex in the future development of CD-based selectively guided tumor therapy.

## Methods

### Antibodies and reagents

The characteristics of the scFvGO used in this study as scFv irrelevant antibody were previously described [17]. Anti-Flag M2 and anti-polyhistidine antibodies were purchased from Sigma (St Louis, MO, USA). The goat anti-mouse HRP-conjugated polyclonal antibody was purchased from Dako (Denmark, EU). 5-Fluorocytosine (5-FC) and 5-Fluorouracil (5-FU) were purchased respectively, from Sigma and Mayne Pharma (Naples, Italy, EU)

### Vector construction

Complete yCD gene sequence [18] was amplified by PCR from cDNA inserted in pACCMV 115. The sense primer was: *BamyCD *5'-CGA ATT GGA TCC ATG GTG ACA GGG GGA-3', containing BamHI restriction site and the sequence coding for first five amino acid of yCD. The antisense primer was: *ESyCD *5'-ATCC GAT ATC GTC GAC CTC ACC AAT ATC TTC-3' containing the sequences encoding for the end part of yCD and SalI restriction enzyme.

PCR was performed using Pwo enzyme (Roche Diagnostics; IN, USA) and the resulting PCR fragment was agarose-purified using the High Pure PCR Product Purification Kit (Roche). Then it was digested with restriction enzymes *BamHI *and *SalI*, and cloned into the plasmid pQE30Xa (Qiagen; Milan, Italy, EU), containing 6 × His tag sequence for protein purification. The clone was sequenced by Biofab Research SRL (Rome, Italy, EU).

### Expression and purification

TG1 *E. coli *(*supE hsd*Δ5 *thi *Δ(*lac-proAB*) F' [*traD*36 *proAB*+ *lacIqlacZ*ΔM15]) cells trasformed with plasmid pQE30Xa yCD were grown in 100 ml 2 × TY broth supplemented with 100 μg ml^-1 ^ampicillin and 0.1% glucose in a 37°C shaker until OD_600 _= 0.6. Isopropyl-β-D-thiogalactopyranoside (IPTG) (Sigma) was added to a final concentration of 1 mM. Cells were harvested 3 h later, centrifuged at 10,000 rpm for 20 min at 4°C and lysed by sonication in lysis buffer (50 mM NaH_2_PO_4_, 300 mM NaCl, 10 mM imidazole, pH 8). The yCD protein was purified by affinity chromatography on Ni-NTA resin (Qiagen), using native protocol according to the manufacture instructions. Protein concentration was determined with Fernandez-Patron method. The purified yCD protein was dissolved in PBS, aliquoted and stored at -80°C.

### NMR

^19^F NMR analyses were performed on BRUKER AVANCE spectrometer (Bruker BioSpin GmbH – Rheinstetten – Germany) operating at 9.4 T. The spectra were acquired at 25°C with a pulse angle of 60°, interpulse delay of 2 s and 64 transients. In order to compensate for partial magnetic saturation effect, the correction factors were determined by comparing the measured peak areas with those obtained at equilibrium (flip angle 90°, interpulse delay 30 s). At the end of reaction the concentration of 5-FU was determined by adding a known amount of the drug. Spectral analyses were performed utilizing the XWIN-NMR BRUKER suite. ^19^F-MRS of 3,5 μmoles of 5-FC dissolved in 700 ul D_2_O saline buffer was considered the time 0 of the reaction and after 70 μl of 25 μg/ml yCD enzyme were added. The reaction was followed during 1 h and 30 min. To verify the complete conversion of 5-FC to 5-FU the last spectrum was acquired at 3 h and 15 min.

### ETH-2 library

The ETH-2 synthetic human recombinant antibodies library consists of a large array (more than 10^9 ^antibody combination) of scFv polypeptides displayed on the surface of M13 phage [14]. It was built by random mutagenesis of the CDR3 of only three antibody germline gene segments (DP47 for the heavy chain, DPK22 and DPL16 for the light chain). Diversity of the heavy chain was created by randomizing four to six position, replacing the pre-existing position 95–98 of the CDR3. The diversity of the light chain was created by randomizing six position (96–101) in the CDR3 [15].

### Selection of yCD protein specific antibodies from ETH-2 library

Immunotubes (Nalge Nunc International; NY, US) were coated overnight (ON) at room temperature (RT) with purified yCD in PBS at the concentration of 10 μg ml^-1^. After panning, performed according to Ascione et al. [17], phages were eluted with 1 ml of 100 mM triethylamine, and the solution was immediately neutralized by adding 0.5 ml of 1 M Tris-HCl pH 7.4. Eluted phages were used to infect TG1 *E. Coli *cells and amplified for the next round of selection. Briefly, 50 ml of 2 × TY with 100 μg/ml ampicillin and 1% glucose (2 × TY-amp-glu) were inoculated with enough bacterial suspension to yield an OD_600 nm _≅ 0.1. The culture was grown to OD_600 nm _= 0.4–0.5 and infected with K07 helper phage at a ratio of around 20:1 phage/bacteria. The rescued phages were concentrated by precipitation with PEG 6000 and used for the next round of panning. For soluble scFv preparation, cloned E. coli cells were grown for 2 h at 37°C in 180 μl of 2 × TY-ampicillin (100 μg ml^-1^) and 0.1% glucose in 96-well plates and induced with 50 μl of 2 × TY-6 mM IPTG. The following day the plates were spun down at 1800 g for 10 min at 4°C and the supernatants containing soluble scFv were recovered and tested for specific yCD recognition in ELISA.

### ELISA

96-well ELISA plates were coated ON with 50 μl/well of 10 μg ml^-1 ^purified yCD in PBS at 4°C. Next day a blocking solution, 2% non-fat milk in PBS (2% MPBS) was added and after 2 h the plates were washed with PBS containing 0.05% Tween 20 (TPBS). Plates were incubated for 2 h at RT with 50 μl of supernatants containing soluble scFv antibodies, anti-Flag M2 antibody and anti-mouse HRP-conjugated antibody. All antibodies were resuspended in 2% MPBS.

The reaction was developed using 3,3'-5,5'-tetramethylbenzidin BM blue and POD substrate soluble (Roche Diagnostics) and stopped by adding 50 μl of 1 M sulfidric acid. The reaction was detected with an ELISA reader (BIORAD; CA, USA) and the results were expressed as OD, i.e. the absorbance per unit length, were absorbance (A) is calculated as A = A (450 nm) – A (620 nm).

### DNA characterization and sequences

Plsmidic DNA encoding for selected scFvs were digested by specific endonucleases and CDR3 regions were sequenced with an automated DNA sequencer (M-Medical/Genenco, Pomezia Italy) using fdseq1 (5'-GAA TTT TCT GTA TGA GG-3') and pelBback (5'-AGC CGC TGG ATT GTT ATT AC-3') primers.

### Soluble scFv purification

The clone scFvH5, was cultured for large-scale scFv production. TG1 *E. coli *infected cells were cultured at 30°C in 2 × TY containing 100 μg ml^-1 ^ampicillin and 0.1% glucose up to OD_600 _= 0.5. After induction of antibody expression by adding 1 mM IPTG to culture, cells were incubated ON at 30°C. Then, the bacterial culture was centrifugated and antibody containing supernatant collected. Antibody fragments were precipitated with ammonium sulfate and dialyzed in PBS. His-tagged scFv fragments were purified by immobilized metal affinity chromatography using Ni^2+^-nitriloacetic acid agarose (Qiagen). ScFv fragments were eluted with 250 mM imidazole in PBS, dialyzed, ELISA tested for specific antigen recognition, and stored at -80°C.

### SDS-PAGE and Western Blot analysis

Purified yCD protein was analyzed on 12% SDS PAGE gel under reducing conditions. Gel was either stained with Fernandez-Patron method or blotted electrophoretically to nitrocellulose membrane, which was blocked in 5% MPBS and then washed three times for 10 min in PBS. For detection of yCD protein, the membrane was incubated either with anti-polyhistidine antibody or with soluble scFvH5. In the first case the membrane was incubated for 2 h with anti-polyhistidine antibody 1:1000 in 2% M/PBS and washed three times with PBS. In the other, the membrane was incubated for 2 h with soluble scFvs, washed with PBS containing 0.05% Tween 20 and incubated again with an anti-Flag M2 mouse antibody 1:1000 in 2% MPBS for 1 h at RT. In both cases specific binding was detected by HRP-conjugated Goat anti-mouse antibody 1:1000 in M/PBS 2% for l h at RT. After 3 washings in 2% M/PBS, the bound antibodies were visualized with DAB buffer obtained by dissolving one tabelet (10 mg) of 3,3'-diaminobenzidine (Sigma) in 20 ml of PBS and 3 μl of hydrogen peroxide 30%, for 3 min. The reaction was stopped with H_2_O.

### Determination of yCD activity

The deamination activity of purified yCD was measured by monitoring conversion of 5-FC to 5-FU in spectrophotometric studies. In 0.5 ml quartz cuvette, 250 μl of 1 μg ml^-1 ^yCD was added to solution of 0.36 mM of 5-FC. The reaction was followed for 30 min by an UV/Vis spectrophotometer (Beckman DU-64, Beckman Coulter S.p.A., CA, USA) which registered absorbance values every 30 seconds. The absorbance variation was measured at 265 nm, wavelength of the 5-FU maximum UV absorption according to Nishiyama et al., 1985 [17]. Absorbance values were calculated as A_265 _(t) - A_265 _(t_0_), (t_0 _= 0 min); the values were converted in concentration of formed 5-FU, dividing absorbance values by 5-FU molar extinction coefficient at 265 nm (ε_265_). The calculated 5-FU ε_265 _was 7 mM^-1 ^cm^-1^. Initial velocity of the enzyme was calculated as ΔA_265 _min^-1 ^or as Δ[5-FU] min^-1^in the first 9 min when the reaction had linear trend.

The same procedures were used in order to examine eventual inhibition of yCD activity occurred in presence of scFvH5. Briefly, 5 μl of 200 μg ml^-1 ^purified scFvH5 solution were added into the cuvette with yCD and 5-FC. Parallel experiments were performed in presence of the irrilevant scFvGO antibody.

### Cytotoxic assay

The ability of purified yCD protein to convert 5-FC into 5-FU was tested in an vitro cell sytem. The human colon adenocarcinoma LoVo cells were maintained in a basic medium (BM) constituted by RPMI 1640 (EuroClone S.p.A; PV, Italy, EU) supplemented with 10% fetal bovine serum (EuroClone) and 1% penicillin-streptomycin in humidified atmosphere with 5% CO_2 _at 37°C.

In a cell growth inhibition assay 2500 cells/well were seeded into 96-well microtiter plates (Corning Cable Systems SRL, Turin, Italy, EU) in BM containing 2.5 μg ml^-1 ^of yCD and different concentrations of 5-FC. The plates were incubated at 37°C for 4 days and cell viability was evaluated by WST-1 assay (Takara, VinciBiochem, Vinci, Florence, Italy, EU).

As positive and negative controls different concentrations of 5-FC and 5-FU alone were used in identical in vitro conditions. A cell growth inhibition assay was also used in order to determine whether the binding with the specific scFvH5 antibody affects yCD enzyme function.

In this experiment LoVo cells (2500 cells/well) were seeded in 96-costar plates in BM containing 2.5 μg ml^-1 ^of yCD and 10 μg ml^-1 ^of 5-FC in presence of scFvH5 or scFvGO antibodies at concentrations ranging from 0.1 to 10 μg ml^-1^. All results were represented as the mean of triplicate samples.

## Abbreviations

5-FC: 5-fluorocytosine; 5-FU: 5-fluorouracil; yCD: yeast cytosine deaminase; scFv: single chain fragment variable; GO: glucose oxidase; mAb; monoclonal antibody

## Competing interests

The authors declare that they have no competing interests.

## Authors' contributions

AM carried out selection of the ETH-2 library against yCD and contributed to the genetic, molecular, and immuno-biochemical characterization of scFvs.SZ carried out expression, production and purification of yCD protein and participated to biopanning selection of the ETH2 library, and biochemical characterization of the antibodies to yCD.MF, AA and MG actively participated to yCD purification and biopanning procedures for scFv isolation and selection. GC and FS with the supervision of FP conceived, promoted and carried out NMR experiments for the functional assay of the yCD enzyme. MT carried out the cell culture experiments including the testing of the cell growth and viability in presence of drug. MC conceived and promoted the approach with the ETH-2 phage library to select specific scFv human antibodies against soluble yCD protein. Furthermore, MC participated in the design and coordination of the entire research project. All authors have read and approved the final version of the manuscript.
